# The Structure of the Mitotic Spindle and Nucleolus during Mitosis in the Amebo-Flagellate *Naegleria*

**DOI:** 10.1371/journal.pone.0034763

**Published:** 2012-04-06

**Authors:** Charles J. Walsh

**Affiliations:** Department of Biological Science, University of Pittsburgh, Pittsburgh, Pennsylvania, United States of America; University of Oklahoma Health Sciences Center, United States of America

## Abstract

Mitosis in the amebo-flagellate *Naegleria pringsheimi* is acentrosomal and closed (the nuclear membrane does not break down). The large central nucleolus, which occupies about 20% of the nuclear volume, persists throughout the cell cycle. At mitosis, the nucleolus divides and moves to the poles in association with the chromosomes. The structure of the mitotic spindle and its relationship to the nucleolus are unknown. To identify the origin and structure of the mitotic spindle, its relationship to the nucleolus and to further understand the influence of persistent nucleoli on cellular division in acentriolar organisms like *Naegleria*, three-dimensional reconstructions of the mitotic spindle and nucleolus were carried out using confocal microscopy. Monoclonal antibodies against three different nucleolar regions and α-tubulin were used to image the nucleolus and mitotic spindle. Microtubules were restricted to the nucleolus beginning with the earliest prophase spindle microtubules. Early spindle microtubules were seen as short rods on the surface of the nucleolus. Elongation of the spindle microtubules resulted in a rough cage of microtubules surrounding the nucleolus. At metaphase, the mitotic spindle formed a broad band completely embedded within the nucleolus. The nucleolus separated into two discreet masses connected by a dense band of microtubules as the spindle elongated. At telophase, the distal ends of the mitotic spindle were still completely embedded within the daughter nucleoli. Pixel by pixel comparison of tubulin and nucleolar protein fluorescence showed 70% or more of tubulin co-localized with nucleolar proteins by early prophase. These observations suggest a model in which specific nucleolar binding sites for microtubules allow mitotic spindle formation and attachment. The fact that a significant mass of nucleolar material precedes the chromosomes as the mitotic spindle elongates suggests that spindle elongation drives nucleolar division.

## Introduction

Acentrosomal mitosis is common in protists and plants. In many cases, the assembly of the spindle microtubules is thought to be organized around the chromosomes. The organization of the microtubules into anti-parallel bundles is believed to be driven by motor proteins (reviewed in [1]). Acentrosomal mitosis in organisms with closed mitosis (the nuclear membrane does not break down) is less well understood.

While nucleoli disassemble during mitosis in most mammalian cells, many organisms divide with persistent nucleoli (reviewed in [2]). In amebae of the genus *Naegleria* mitosis is closed and the nucleolus persists throughout the cell cycle. The *Naegleria* nucleolus contains approximately 4,000 copies of a small circular plasmid. Each plasmid codes for 5.8S, 18S and 28S rRNA. Because the nuclear chromosomes do not contain copies of the rDNA, nucleolar division is essential if each daughter nucleus is to obtain a suitable portion of the nucleolar plasmids [3], [4]. The nucleolus divides as the mitotic spindle elongates (reviewed in [5]), [6], but the origin and structure of the *Naegleria* mitotic spindle are unknown. It is unclear how and where these spindle microtubules assemble and how they are organized inside the intact nuclear envelope in the absence of detectable centriole-like structures or MTOCs (microtubule organizing centers) [7].

The mechanism responsible for the division of the large nucleolus is also unknown. Some early studies suggested a role for the mitotic spindle in nucleolar division but the techniques then available were not sufficient to examine this question in detail. Later studies by electron microscopy occasionally showed spindle microtubules in the same plane as material thought to be of nucleolar origin but they did not allow a determination of the structure or extent of the association between the nucleolus and the mitotic spindle [6]–[8].

In the current work I have used antibodies against *Naegleria* nucleolar proteins and spindle microtubules, combined with 3D reconstructions using confocal microscopy, to examine the detailed organization of the nucleolus and the mitotic spindle as *Naegleria* amebae undergo mitosis. These data demonstrate that the first detectable spindle microtubules are seen on or within the surface of the nucleolus. The intimate association between the mitotic spindle and the nucleolus persists throughout mitosis. The data suggest that the nucleolus plays an important role in the formation of the mitotic spindle and that elongation of the mitotic spindle in turn plays a role in the division of the nucleolus.

## Results and Discussion

*Naegleria* amebae lack microtubules except during mitosis or as they differentiate into flagellates. In flagellates, microtubules are confined to the cytoplasm [7], [9]. In log phase cultures, not induced to differentiate, any tubulin staining readily identifies mitotic cells. This allows the easy visualization of each mitotic stage without the necessity of synchronizing cultures thus preserving normal cellular morphology. Because closed mitosis lacks some of the readily recognized transitions of open mitosis, most notably nuclear membrane breakdown, there have been a number of discussions of the appropriate terminology for the stages during *Naegleria* mitosis; reviewed in [5]. In this work, I have used the usual mitotic stages taking the first appearance of microtubules within the nucleus as the onset of prophase. Subsequent mitotic stages are readily recognized based on the changes in DNA distribution and conform to the standards for the mitotic phases common to most eukaryotes. In the current work 16% of early-log phase amebae showed some microtubule staining, all of which was confined to the nucleus (N = 200). Of the cells examined in full optical sections (N = 120), 43% were identified as prophase, 37% as metaphase, 12% as anaphase, and 8% as telophase.

Mitotic cells were stained using monoclonal antibodies against three different components of the nucleolus, as well as with a monoclonal antibody against α-tubulin. The nucleolar protein BN46/51 (identified by the monoclonal antibody BN5.1) is a part of the Granular Component (GC) of the nucleolus [10]. BN46/51 is known to bind to two other nucleolar components in *Naegleria*, the DE6 and AH6 antigens [10], [11]. While BN46/51 has not been identified in other cell types, BN46/51 does bind to both mammalian and yeast nucleoli, presumably because of the presence of proteins similar to the DE6 and AH6 antigens in these cells [11]. When the two BN46/51 subunits, 46 kD and 51 kD, were expressed separately in yeast, both subunits were specifically targeted to the nucleolus [11]. The nucleolar proteins identified by the monoclonal antibody DE6 are part of the Dense Fibrillar Component (DFC) of the nucleolus. In *Naegleria*, the DFC and GC are distributed throughout the compact single nucleolus [10]. A third monoclonal antibody, AH6, reacts with both the GC and the DFC of *Naegleria* nucleoli. Lesser amounts of the AH6 antigen are also found in the nucleoplasm and the cytoplasm, most likely reflecting association with ribosomes [10]. Both the AH6 and the DE6 antibodies bind to mammalian and yeast nucleoli. The DE6 antigen in yeast has been identified as NOP1 (fibrillarin) [10], [11].

**Figure 1:**
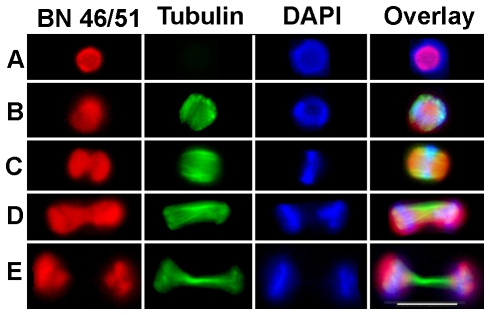
Stages of Mitosis in Naegleria Revealed by Staining for α-Tubulin, BN46/51 and DAPI. Images were obtained by conventional epifluorescence microscopy. A. Interphase; B. Prophase; C. Metaphase; D. Anaphase; E. Telophase. Bar = 10 µm. Red, BN46/51; Green, α-tubulin; Blue, DAPI.

The general relationships between mitotic spindle microtubules, the nucleolus and the distribution of DNA during *Naegleria* mitosis are illustrated in Fig. 1. Microtubules are first seen as overlapping the nucleolus as imaged with BN5.1. As cells progress into metaphase, the microtubules become organized into a band perpendicular to the chromatin metaphase plate confined within the nucleolus. As cells progress through anaphase, the band of microtubules elongates leading to separation of the nucleolus into two broad masses at each end of the telophase spindle. Microtubules appear to remain coincident with the nucleolar material throughout mitosis. A similar distribution was seen using monoclonal antibodies DE6 and AH6.

**Figure 2:**
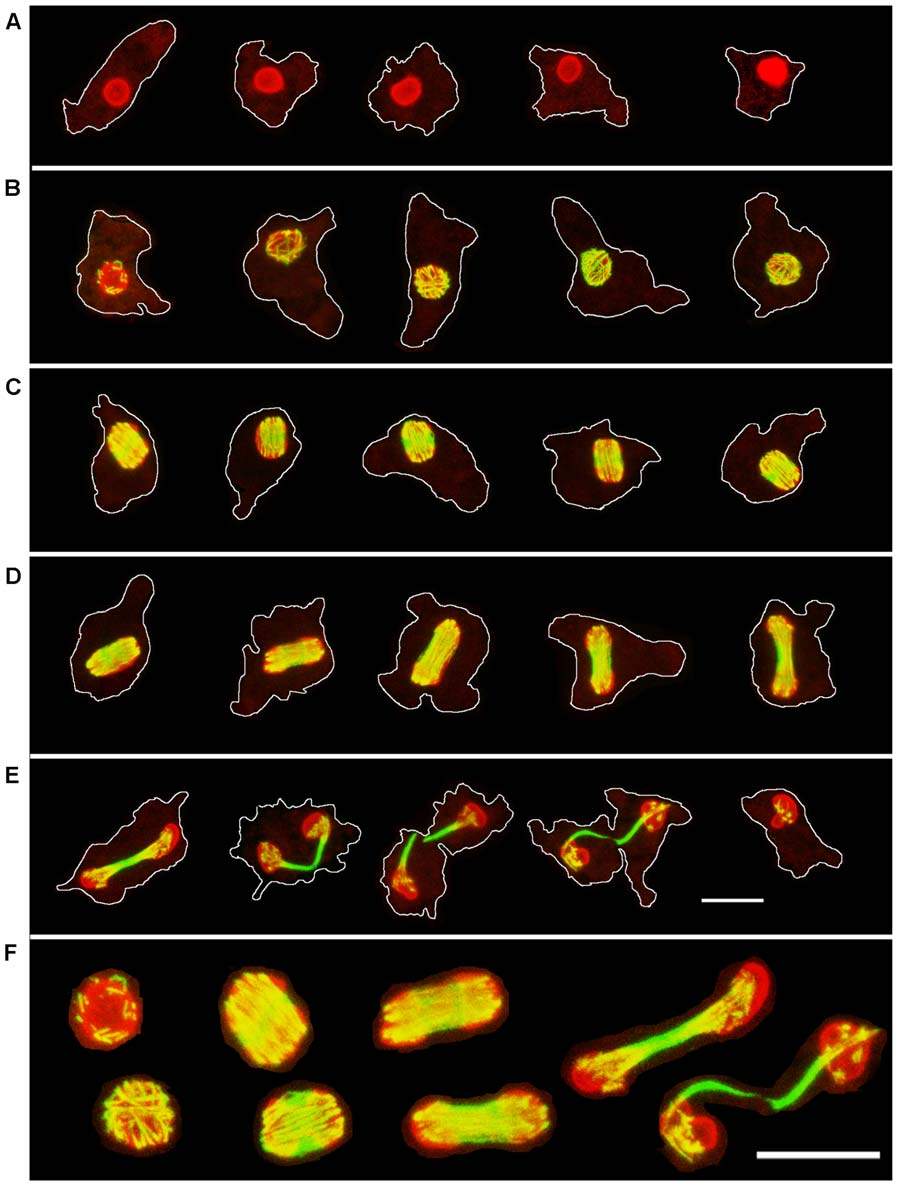
Confocal Microscopy of Mitotic Cells Stained for α-Tubulin and the Nucleolar Protein BN46/51. Five examples of progressively later stages of each mitotic stage are presented. Each image is a Z-axis projection of 0.25 µm optical sections through an individual cell. The cell periphery is outlined in white. A. Interphase; B. Prophase; C. Metaphase; D. Anaphase; E. Telophase. F. Selected nuclei of, from left to right, Prophase, Metaphase, Anaphase, and Telophase. Bar = 10 µm. Red, BN 46/51; Green, α-tubulin.

In order to obtain a better understanding of the structure of the mitotic spindle and it relationship to the nucleolus, cells were imaged using confocal microscopy. The data presented in Fig. 2, obtained using the BN5.1 antibody, are representative samples from the detailed analysis of more than 120 individual mitotic cells optically sectioned throughout the cell at 0.25 µm intervals. The images were produced by projecting 0.25 µm confocal sections along the Z-axis. These data reveal, for the first time, the arrangement of the spindle microtubules during mitosis in *Naegleria* and show the evolving relationship between the spindle and dividing nucleolus in this organism.

During interphase no microtubules were seen in amebae and the core of the nucleolus was diminished, Fig. 2A, consistent with previous reports [5], [10]. Microtubules typically first appeared as short isolated strands or “V” shaped bundles on the surface of the nucleolus, Fig. 2B and Movie S1. As the number of microtubules increased they formed a cage surrounding the nucleolus, Figs. 2B. The three-dimensional reconstruction in Movie S1 clearly shows that the vast majority of the spindle microtubules are surrounded by nucleolar material. This point is further supported by the reconstructions of subsequent stages provided in Movies S2, S3 & S4.

With the approach to metaphase, microtubules became organized into parallel bundles perpendicular to the future metaphase plate, Fig. 2C and Movie S2. It has been suggested that the formation of this type of dense bundle of microtubules, driven by microtubule motor proteins, is the essential feature necessary for the successful formation of a functional mitotic spindle regardless of the source of the microtubules; whether from a centrosomal organizing center, chromatin or kinetochores [1], [12].

A distinct gap in the distribution of nucleolar proteins developed with the formation of the metaphase plate. The mitotic spindle became barrel shaped without a clear point of origin. The spindle microtubules were seen to terminate within the nucleolar masses, Fig. 2C and Movie S2. With the onset of anaphase, the nucleolar masses tracked with the elongation of the mitotic spindle. Relatively little residual nucleolar material is seen in the widening gap between the chromosomes. In almost all cases the nucleolar proteins extend beyond the ends of the elongating spindle, Fig. 2D and Movie S3, indicating the microtubules are embedded within the nucleolar material throughout mitosis.

The fact that in anaphase the mitotic spindle did not contract at the ends is not typical, even in acentrosomal mitosis. Usually there is a contraction of the ends of the spindle forming two prominent loci of focused minus ends during anaphase even when DNA coated beads are used to activate microtubule assembly [1]. The absence of this contraction suggests that the distal ends of the mitotic spindle are constrained, most likely by interaction with nucleolar components.

In telophase, the chromosomes were usually seen in a gap in the spindle microtubules. An irregular mass of short microtubules was seen at the distal ends of the spindle while a dense band of microtubules extended between the new nuclei, Fig. 2E and Movie S4. This band is the region referred to as the interzonal body in earlier work [5]. The gap that developed in the mitotic spindle during telophase in the region containing the chromosomes might represent the decondensation of the chromosomes with the resulting loss of microtubule binding sites, Fig. 2E and Movie S4. Dense fragments of microtubules were seen to persist in some daughter nuclei after the band of microtubules between the daughter nuclei had broken and the chromosomes had dispersed, Fig. 2E.

The DE6 antigen (data not shown) shows a similar distribution to that in Fig. 2. The optical sectioning of more than 120 individual mitotic cells showed no microtubules in the cytoplasm or nucleoplasm. All microtubules were seen either at the surface of the nucleolus or embedded within the nucleolus throughout mitosis.

It has been suggested that the interzonal body represents nucleolar material (see for example [5]). However, there was no evidence of either the BN5.1 or the DE6 antigens in this region during telophase in these studies. A low level of the AH6 antigen was seen between the ends of the elongating spindle but this is the same low level seen throughout the cell. This is thought to represent staining associated with ribosomes.

An actin-based system has been suggested as being responsible for nuclear division in *Amoeba proteus* [13]. No actin was detectable in the mitotic nucleus of *Naegleria* using an antibody specific to *Naegleria* actin, phaloidin or DNase I (see especially Fig. S2 in [14]). However, this question must remain open because there are many challenges in detecting actin or myosin in the mitotic spindle region of even well studied cell types [15].

To more accurately determine the location of the earliest detectable spindle microtubules, detailed analysis of the signals for tubulin and the nucleolar proteins BN46/51 and DE6 were carried out on confocal sections. It has been customary to use the yellow color produced by overlaying red and green labeled markers to determine such co-localization. However, when a series of known red and green intensities were examined in overlays it was evident that this technique is of limited accuracy. Only when both colors were near maximum intensity (255 in an 8 bit image) did an overlay by the screen function in Photoshop produce a distinct yellow color. Even at a ratio of 1∶1.3 (200∶255), the results were either orange or yellow-green depending on the stronger color. In addition, the resulting color was different depending on which color was placed on top.

Therefore, a different approach was developed in order to gain a quantitative understanding of the relative distribution of the early spindle microtubules and the nucleolar proteins. The intensity of the nucleolar label of each individual pixel in an optical section was used to determine if the corresponding tubulin pixel would be displayed. To provide a stringent test of the co-localization, tubulin staining for a given pixel was displayed only if the corresponding nucleolar protein pixel reached a given minimum intensity. Two tests of co-localization were carried out. In the first instance, the nucleolar antigen staining was required to equal or exceed 25% (64/255) of the maximum possible nucleolar intensity. In the second test, the nucleolar antigen staining was required to equal or exceed 50% of the maximum possible intensity (128/255). If the given level of nucleolar protein intensity was not reached, the corresponding tubulin pixel was changed to black. In all cases, care was taken to make sure that fluorescence intensity did not exceed the dynamic range of the detectors

**Figure 3:**
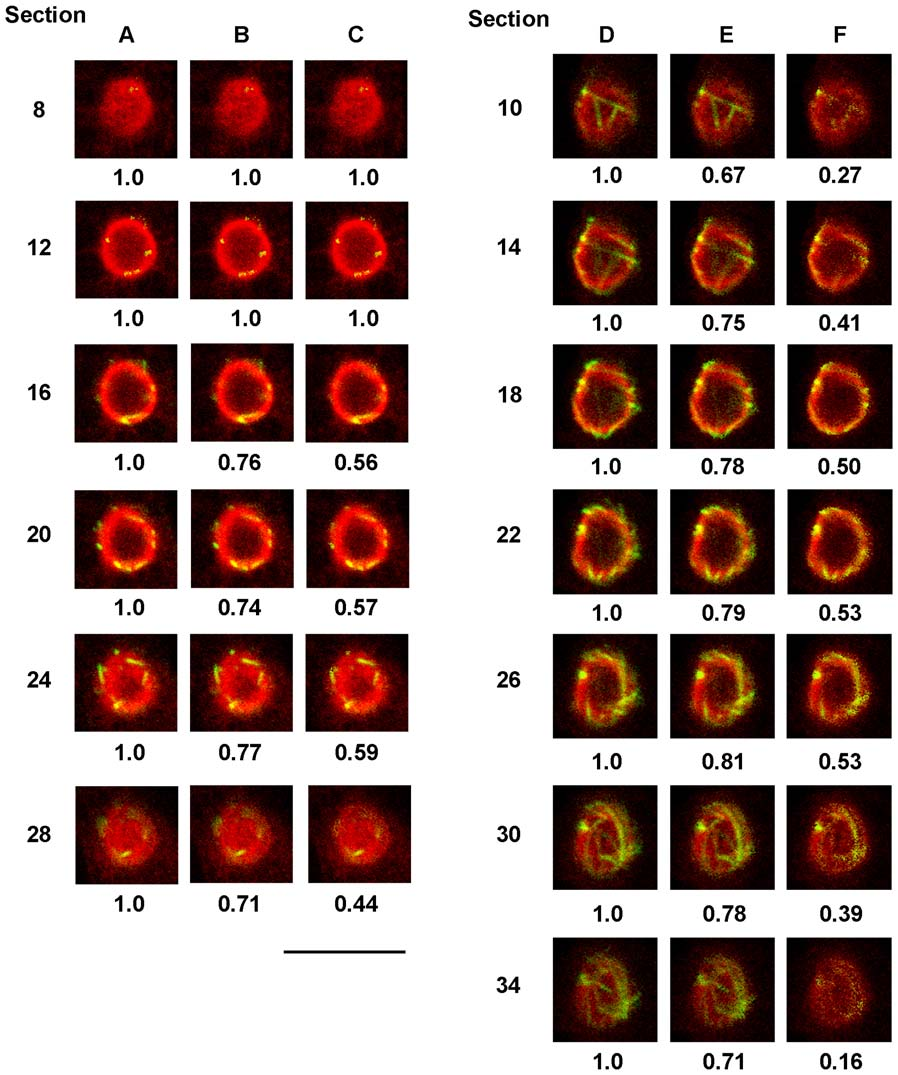
Analysis of the Co-localization of Tubulin and the Nucleolar Protein BN46/51 During Prophase. Single 0.25 µm optical sections at 1.0 µm intervals are presented for two different cells. Columns A and D; raw data. Columns B and E; tubulin pixels are only displayed if the corresponding nucleolar protein pixel equaled or exceeded 25% of the maximum possible intensity. Columns C and F; tubulin pixels are only displayed if the corresponding nucleolar protein pixel equaled or exceeded 50% of maximum possible intensity. Numerical values are the summed fraction of tubulin intensity remaining in a given section after the subtraction. Red, BN 46/51; Green, α-tubulin. Bar = 10 µm.

Figs. 3A & 3D present confocal sections, at approximately 1 µm intervals, of two early prophase cells stained with BN5.1 and anti-α-tubulin. The tubulin staining remaining in each section, after removal of tubulin that is not coincident with BN46/51 equal to or greater than 25% of the maximum possible intensity, is shown in Figs. 3B & 3E. The numerical values under each section are the fraction of the total tubulin staining remaining in that section after the subtraction of tubulin not coincident with the tested BN46/51 intensity. It is evident that in all sections, except for the most peripheral, 70% or more of the tubulin intensity is associated with BN46/51, the intensity of which equaled or exceeded 25% of the maximum possible intensity. A more stringent test, which removed tubulin staining that was not coincident with BN46/51 staining the intensity of which equaled or exceeded 50% of the maximum possible nucleolar staining is presented Figs. 3C and 3F. In this more stringent test, 50% or more of the tubulin intensity was still retained in all but the peripheral sections. It is worth noting that even when the BN46/51 staining, coincident with tubulin, equaled or exceeded 50% of its maximum possible intensity, a significant fraction of the tubulin pixels retained are still imaged as green rather than yellow. The same analysis using the DE6 nucleolar antigen produced essentially the same results. By following individual areas of microtubule staining in sequential sections, it is also apparent that they remain, for the most part, entirely within the nucleolus. This analysis allows us to conclude that even during the earliest stages of prophase the majority of the spindle tubulin is coincident with both the BN46/51 and DE6 nucleolar proteins. It is also important to note that there is no microtubule staining outside of the periphery of the nucleolus in these cells.

The three-dimensional reconstruction of an early prophase nucleus, Movie S1, also clearly shows that the vast majority of the spindle microtubules are surrounded by nucleolar material at this time. This point is further supported by the reconstructions of subsequent stages provided in Movies S2, S3 and S4. The restriction of the spindle termini within the nucleolus and the separation of the nucleolar material along with spindle elongation suggest specific binding sites for the ends of spindle microtubules within the nucleolus.

It is important to distinguish between mechanisms that may target tubulin polymerization to the nucleolus (MTOCs) and mechanisms which might crosslink spindle microtubules to nucleolar components. Only the later would be likely to allow the mitotic spindle to drive nucleolar division. At present no specific interactions between nucleolar proteins and spindle microtubules have been characterized. The BN46/51 nucleolar protein is found concentrated in a small spot over the basal bodies in the cytoplasm of flagellates, the point of origin of the cytoplasmic microtubules (thought to be an MTOC) [16]. Preliminary, unpublished data, suggested that BN46/51 might bind to cytoplasmic microtubules based on binding to purified tubulin [16] but this result has not proved reproducible. In addition, BN46/51 was not seen associated with the cytoplasmic microtubules, which are only present in flagellates [16]. The recent description of a mitosis specific form of α-tubulin [17] leaves open the possibility of a specific interaction between spindle microtubules and one or more nucleolar proteins. This possibility remains to be tested.

In other acentrosomal systems, microtubules have been shown to assemble around chromosomes [12]. Either the many small nuclear chromosomes or the thousands of nucleolar plasmids [3], [4] could serve this function in *Naegleria*. In either event, these chromosomes would be expected to be directly adjacent to the surface of the nucleolus where spindle microtubules are first detected.

The data presented here demonstrate that in *Naegleria*, the mitotic spindle microtubules first appear as short rods in close association with the nucleolar surface. These microtubules form an irregular cage surrounding the nucleolus and then reorganize into a broad band, spanning the metaphase plate that is embedded within the nucleolus. This reorganization of spindle microtubules into a band or bundle is similar to the organization of mitotic spindles, driven by microtubule motor proteins, described in other acentrosomal cells [12]. However, as far as I am aware, the close association of the mitotic spindle with the nucleolus is unique to this study and adds to our understanding of nucleolar division in acentrosomal organisms with a closed mitosis and persistent nucleoli. The extension of the mitotic spindle during anaphase is a plausible explanation for the source of nucleolar division given the fact that the spindle microtubules are imbedded within the nucleolus. Testing this hypothesis will require additional studies to see if spindle microtubules make specific interactions with one or more nucleolar components and to see if disrupting such contacts blocks nucleolar division. The extension of the large nucleolus might in turn help drive nuclear division. This view is consistent with the observation that the telophase nucleolus is seen in close apposition with the nuclear membrane by electron microscopy [6], [8].

The ability to reassemble nucleolus-like particles, which contain 40% of the nucleolar DNA, RNA, and protein, from soluble extracts of *Naegleria* nucleoli should provide a system in which both the organization of the nucleolar plasmids and the binding of microtubules can be examined *in vitro* [10]. Combined with the recent analysis of the *Naegleria* genome [18], such an *in vitro* system would help advance not only our understanding of mitosis in *Naegleria* but our understanding of the evolution of mitosis in early eukaryotes.

## Materials and Methods

Amebae of *Naegleria pringsheimi* strain NB-1, ATCC # PRA-212 (formerly *Naegleria gruberi* [19]) were grown on NM agar with a lawn of *Klebsiella pneumoniae* as previously described [20]. Early log-phase amebae were harvested from plates in which the bacterial lawn had not begun to clear. Amebae and the accompanying bacteria were gently resuspended in 5 ml of 2 mM Tris (2 mM Tris-HCl, pH 7.6) warmed to the incubation temperature (34°C), using a glass spreader. The suspension was immediately fixed by addition to an equal volume of room temperature 7.4% formaldehyde in 50 mM sodium phosphate, pH 7.2. Formaldehyde, prepared from paraformaldehyde, was purchased as a 32% solution (Electron Microscopy Sciences, Hatfield, PA).

After 30 minutes at room temperature, fixed cells were embedded in low melting point agarose as previously described [14]. The agarose squares were transferred to PBS (0.15M NaCl, 40 mM sodium phosphate, pH 7.2) in 2.5 cm diameter by 1 cm deep wells in a block of Teflon® by gentle rinsing with PBS. All washing and staining was carried out in these wells by removing fluid using gentle suction to prevent aspirating the agarose. Fixed cells were permeabilized for 30 minutes in 0.1% Triton-X-100 in PBS at room temperature. After blocking with 2% ovalbumin in 0.1% Tween 20 in PBS at room temperature for 30 minutes, cells were incubated with first antibody for 60 minutes at 37°C or in some cases overnight at 3°C. After washing 4 times with PBS, samples were incubated with second antibody for 60 minutes at 37°C and washed 4 times with PBS. Agarose squares were mounted in triangular wells in 100 mg/ml DABCO (1,4-diazabicyclo-[2.2.2] octane) in 10% PBS, 90% glycerol as previously described [14].

Microtubules were imaged with mouse monoclonal anti-α-tubulin AA4.3, developed against *Naegleria* tubulin [9]. When double-labeling nucleolar proteins and microtubules, microtubules were imaged with the rat anti-tubulin monoclonal YOL1/34 (MCA 78G from AbD Serotec). YOL 1/34 was used at 1∶50 dilution in PBS; all other monoclonal supernatants were used undiluted. Nucleolar antigens were imaged with mouse monoclonal antibodies AH6, DE6 or BN5.1 raised against *Naegleria* nucleolar proteins [10]. AA4.3, AH6, DE6 and BN5.1 are available from the Developmental Studies Hybridoma Bank at the University of Iowa. Second antibodies were goat, anti-mouse IgG, or anti-rat IgG labeled with Alexa dyes (Molecular Probes, Eugene, OR) at a dilution of 1∶200 as described in individual figure legends. DNA was imaged by staining with DAPI at 10 µg/ml in PBS at room temperature for 30 min followed by two 5 min washes with PBS.

Images were obtained with a Nikon Eclipse E800 microscope equipped with a 63×, 1.4 NA oil immersion objective and a BioRad Radiance 2000 confocal system operating in the λ strobe mode. The absence of crosstalk for all combinations of second antibodies was confirmed using appropriate controls. Care was taken to image cells close to the cover slip to insure maximum resolution. Illumination intensity was controlled to insure that all images were within the dynamic range of the detectors.

Images were analyzed using version 175-1C of Image SXM developed by Dr. Steve Barrett (available at www.liv.ac.uk/~sdb/ImageSXM/). Single gray scale images were transferred to Adobe Photoshop 7, converted to RGB color using a linear LUT, and overlaid to produce multicolor figures. Stacks of confocal sections were projected using the brightest point technique in Image SXM. Individual views were processed in Photoshop as above. Image SXM macros for pixel-by-pixel analysis are available from the author.

## Supporting Information

Movie S1 **3D Reconstruction of a Typical Early Prophase Mitotic Spindle and Nucleolus.** The movie was produced by projection around the Y-axis at 10° intervals in Image SXM. Individual views were combined in Photoshop as described above for still images and then assembled in QuickTime Player Pro. Red, Nucleolar protein BN 46/51; Green, α-tubulin.
(MOV)

Movie S2 **3D Reconstruction of a Typical Metaphase Mitotic Spindle and Nucleolus.** The movie was produced as described for Movie S1. Red, Nucleolar protein BN 46/51; Green, α-tubulin.
(MOV)

Movie S3 **3D Reconstruction of a Typical Anaphase Mitotic Spindle and Nucleolus.** The movie was produced as described for Movie S1. Red, Nucleolar protein BN 46/51; Green, α-tubulin.
(MOV)

Movie S4 **3D Reconstruction of a Typical Late Telophase Mitotic Spindle and Nucleolus.** The movie was produced as described for Movie S1 except that the projection was produced around the X-axis. Red, Nucleolar protein BN 46/51; Green, α-tubulin.
(MOV)
